# Supplementary material for: Identification of microRNAs Responding to Aluminium, Cadmium and Salt Stresses in Barley Roots
Source: Plants (Basel). 2021 Dec 14;10(12):2754. doi: 10.3390/plants10122754 (PMC8704135; doi:10.3390/plants10122754)
Supplement: Supplementary file 1 [file plants-10-02754-s001.zip › Supplementary Figures.pdf]

Supplementary Figure(s)

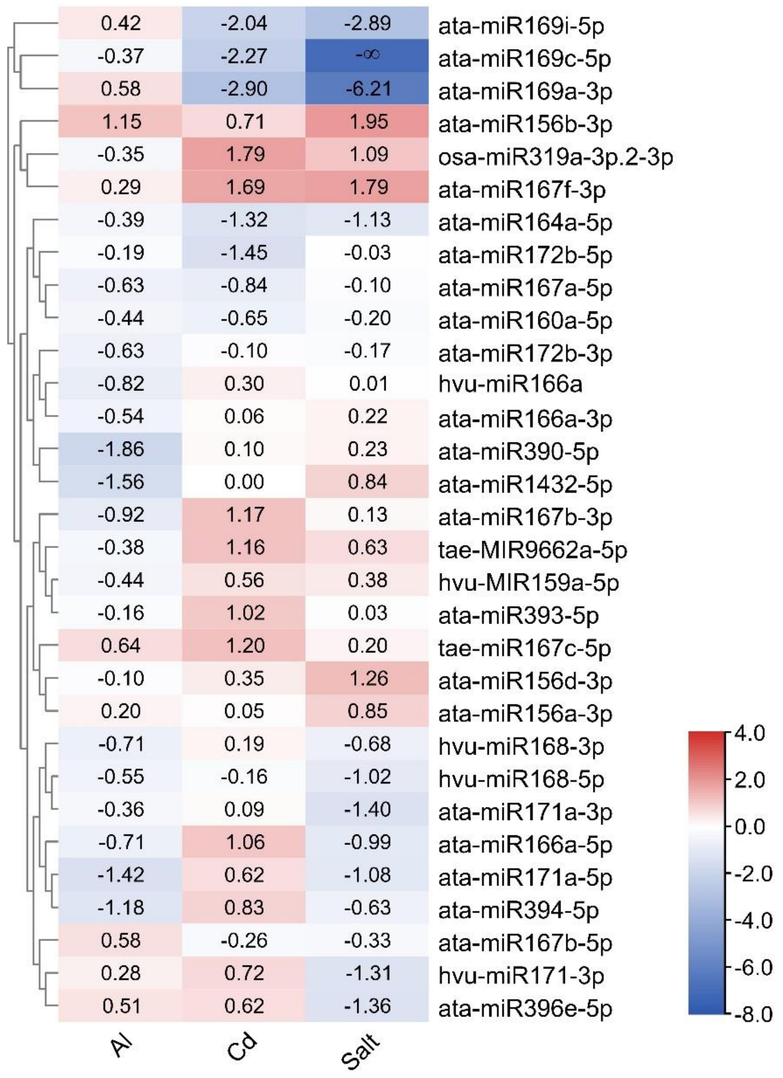

Figure S1. The heatmap of 31 miRNAs in response to Al, Cd and salt stresses. The values represent the fold changes of normalized reads between different treatments (Al, Cd and salt stresses) and their controls. It is calculated as the formula: fold change=log2 (stress reads/control reads). -∞ indicates miRNA expression was not detected in Golden Promise under salt treatment. The "MIR" in miRNA name means the miRNA belongs to new member of known miRNAs.
