# Supplementary material for: Identification of microRNAs Responding to Aluminium, Cadmium and Salt Stresses in Barley Roots
Source: Plants (Basel). 2021 Dec 14;10(12):2754. doi: 10.3390/plants10122754 (PMC8704135; doi:10.3390/plants10122754)
Supplement: Supplementary file 1 [file Response to reviewer 1.pdf › Response to reviewer 1.pdf]

Response to Reviewer #1 Comments

Dear Reviewer 1,

Thank you very much for the careful reviewing and constructive comments. We have revised the manuscript according to your comments. All the comments have been responded point by point as follows. Hopefully the revised version has been much improved in academic quality.

Thank you again for your great support

Best regards
Authors

Major comment: According to the authors, the findings of their study, when combined with the degradome sequencing analysis, reveal that a putative regulatory network of miRNAs is connected with the tolerance of barley roots to Al, Cd, and salt stresses. Overall, manuscript is well written I have some concerns.

Response: Thanks for your positive comments, and we have made the revision based on your comments and suggestions as below.

Comment 1: Abstract must be rewritten with more detail information.
Please start each sentence with full form, no matter you are using abbreviations.

Response: Thanks a lot for your valuable suggestions! We have rewritten the Abstract with more detail information and full form of abbreviations in each sentence is used (Lines 10-26).

Comment 2: Even though the authors provide an extensive literature review of the Introduction, they should enhance and reconstruct it in the introduction section. They should provide the scope of the paper clearly.
In general, there is a repetition of information that might have been omitted.

Response: Thanks for the constructive comments. We have reconstructed the Introduction section and described the objective of the paper clearly in the revised manuscript.

Comment 3: Check the English language of the results and discussion section.
Discuss every section in detail. Most of the sections are left open.

Response: Thank you! We have carefully checked and revised the language in Results and Discussion section and discussed the results in detail.

Comment 4: Update conclusion section in a meaningful way.

Response: A good suggestion! We have revised the conclusion and make it more

meaningful (Line 350-359).
