# Supplementary material for: Identification of microRNAs Responding to Aluminium, Cadmium and Salt Stresses in Barley Roots
Source: Plants (Basel). 2021 Dec 14;10(12):2754. doi: 10.3390/plants10122754 (PMC8704135; doi:10.3390/plants10122754)
Supplement: Supplementary file 1 [file Response to reviewer 2.pdf › Response to reviewer 2.pdf]

Response to Reviewer #2 Comments

Dear Reviewer 2,

Thank you very much for the valuable comments on our manuscript. We have carefully revised the manuscript according to the comments. All the comments have been responded point by point as follows. Hopefully the revised version has been much improved in quality.

Your consideration for this manuscript is highly appreciated.

Sincerely yours,
Authors

Major comment: The aim of this study is to develop a small RNA library in order to identify miRNAs responding to a single or multiple stresses. The paper is well written and deserves to be published.

Response: Thanks a lot for your positive comments, and we have made the revision based on your suggestion as below.

There are some minor suggestions:
Comment 1: The introduction could be focused more on the identification of microRNAs.

Response: Thanks for your suggestion! We have added more information on the identification of microRNAs in the Introduction section (Lines 51-72).
