# Supplementary material for: Identification of microRNAs Responding to Aluminium, Cadmium and Salt Stresses in Barley Roots
Source: Plants (Basel). 2021 Dec 14;10(12):2754. doi: 10.3390/plants10122754 (PMC8704135; doi:10.3390/plants10122754)
Supplement: Supplementary file 1 [file Response to reviewer 3.pdf › Response to reviewer 3.pdf]

Response to Reviewer #3 Comments

Dear Reviewer 3,

Thank you very much for the careful reviewing and constructive comments on our manuscript. We have carefully revised the manuscript according to the comments. All the comments have been responded point by point as follows. Hopefully the revised version has been much improved in academic quality.

Your consideration for this manuscript is highly appreciated.

Sincerely yours,
Authors

Major comment: Title: "Identification of microRNAs responding to aluminum, cadmium and salt stresses in barley roots"
The manuscript describes the identification of 31 new miRNA for Cd, Al, and salt stress in roots of barley (Hordeum vulgare) grown in hydroponic conditions. Specific miRNA (5,4,3) were found for Cd, Al, and salt stress respectively affecting the plant cell wall structure, the jasmonate and ABA signalling pathways. The majority of the other 31 new miRNA were for transcription factors.
The work is interesting for the study of plant stress although the conditions of the experiment are far from a realistic scenario.

Response: Thanks a lot for the valuable comments. The conditions of the experiments are conducted according to the previous studies, such as *Huang et al.* (2020, doi:10.1104/pp.19.00882) for salt stress, *Wu et al.* (2016, doi:10.1104/pp.16.01189) for Cd stress and *Wu et al.* (2020, doi:10.1093/jxb/eraa349) for Al stress. We completely agree with the comment that the conditions of the experiment are far from a realistic scenario. The aim of this study is to identify several miRNAs responding to a single or multiple stresses. To further investigate their functions, we are attempting to obtain the transgenic lines and conduct the experiments both in the lab (hydroponic/pot experiments) and field (salt/Cd/Al affected soil) in the future.

Comment 1: There is one obscure point in the methods section:
Lines 256-260: it appears that the Cd and the salt stress were administered at the same time, while in the whole manuscript they were treated as separately administered. Please clarify this important point.

Response: Thanks for your suggestion! Actually, the three stresses were treated as separately administered to generate miRNA sequencing data because of the different treatment methods for salt, Cd and Al stress. We have made the revision and added the detailed information in the Materials and Methods section (Lines 286-298).

Comment 2: Line 270: For the analysis of the degradosome the authors followed Ma et al 2010, did the Author use the software package CleaveLand to accomplish all the calculations related to the data elaboration?

Response: Yes, you are right! In the study, the software package CleaveLand3.0 was used to accomplish all the calculations related to the data elaboration. Besides, we have cited the reference for CleaveLand (*Addo-Quaye et al.* 2009) in Line 337 (ref.53).

Comment 3: In the section on Results, why didn't the authors produce a heatmap of the up and down-regulated miRNA in the three conditions?

Response: A good suggestion! We have produced a heatmap of the up- and down-regulated miRNA in the three conditions (**Figure S1** in the revised version).

Comment 4: In Table 1, what does the number below the miRNA sequence mean? Please insert their meaning in the table caption.

Response: Thank you! The number actually belongs to the transcript ID of the target gene of miRNA. The abnormal arrangement is due to our improper typesetting of Table 1. We have changed the layout in the revised manuscript (Line 127).

Comment 5: In the Discussion, Line 145-146: this sentence needs a reference.

Response: Thanks for your suggestion, and we have added the related references for the sentence (Lines 162-163).

Comment 6: Line 150: The Authors should be careful when they indicate that some miRNAs are present under double or triple stress. As a fact, the Authors did not perform experiments with actually double or triple stresses, or so they affirm, therefore the miRNA found out are only present in the combination of two and three stress separately, which is a different fact altogether.

Response: You are right! We have revised the less rigorous expression based on your comments (Line 168).

Comment 7: Lines 193-194: Define what ARF stands for when talking about genes

Response: Thanks for your comment! We have defined the ARF in the Discussion section 3.2 (Lines 212-213).

Comment 8: Lines 202-203: this assertion need a reference for backup

Response: Thanks! We have added the reference for the assertion in Lines 221-224.

Comment 9: Lines 211-215: the presence of secondary metabolites such as anthocyanin does not mean that there is oxidative stress going on and that ROS are present. Moreover, the authors did not measure the level of oxidative stress in the plant roots due to the treatment, nor the amount of possible anthocyanin produced into the roots (or even better into the whole plant). Also in Figure 5 the presence of ROS is all hypothetical.

Response: Thanks a lot for the constructive comment, and we have made the revision

on the less rigorous expression and Figure 5 in the new version (Lines 236-238 and Figure 5).

Comment 10: The Conclusion content does not vary from that of the Abstract.

Response: Thanks for your advice! We have rewritten the Conclusion section (Lines 350-359).

Comment 11: There are two blearing spell mistakes, one in the title where Aluminium is misspelled and another in the abstract where at line 10 there is “Barely” instead of barley. In the abstract, the full scientific name of barley, Hordeum vulgare, should have been mentioned.

Response: Thanks! ‘Aluminum’ and ‘Aluminium’ are both commonly used in references, such as ‘Aluminum’ in *Ye et al.* (Plant Cell, 2017, doi: 10.1105/tpc.17.00211) and ‘Aluminium’ in *Delhaize et al.* (Trends Plant Sci., 2012, doi: 10.1016/j.tplants.2012.02.008). In the new version of periodic table of elements (2018.12.01) released by IUPAC (International Union of Pure and Applied Chemistry), Al is ‘**Aluminium**’. Therefore, we have revised ‘Aluminum’ to ‘**Aluminium**’ in the main text of the manuscript. Besides, we have revised the spelling mistake of barley and mentioned the full scientific name of barley (*Hordeum vulgare*) in the Abstract (Line 11).

Comment 12: There are four references that are far too old:
Ref 26 and 27 both from 1993 and 5 and 6 from 1995. I am sure there are more recent references available for the same purposes. Moreover, Ref 8, Su et al., 2014, does not display the Figures in the online version, here again, please find a substitute.

Response: Thank you! We have renewed these four references (Ref. 5, 6, 26, 27) to more recent ones (*Kochian et al.* 2015; *Ahmed et al.* 2016; *Kopittke et al.* 2016; *Dai et al.* 2017) and found a substitute (*Rai et al.* 2019) for Ref. 8, *Su et al.*, 2014.
